# Expression of Salivary and Serum Malondialdehyde and Lipid Profile of Patients with Periodontitis and Coronary Heart Disease

**DOI:** 10.3390/ijms20236061

**Published:** 2019-12-01

**Authors:** Gaetano Isola, Alessandro Polizzi, Simona Santonocito, Angela Alibrandi, Sebastiano Ferlito

**Affiliations:** 1 Department of General Surgery and Surgical-Medical Specialties, School of Dentistry, University of Catania, 95124 Catania, Italy; 2 Department of Economical, Business and Environmental Sciences and Quantitative Methods, University of Messina, 98122 Messina, Italy

**Keywords:** periodontitis, coronary heart disease, malondialdehyde, C-reactive protein, endothelium

## Abstract

Malondialdehyde (MAA) within a lipid pathway has been demonstrated to possess an important role in endothelial function that undergoes periodontitis and coronary heart disease (CHD) development. This study evaluated the impact of periodontitis, CHD, or a combination of both diseases (periodontitis + CHD) on salivary and serum MAA levels. The periodontal and clinical characteristics, serum, and saliva samples were collected from 32 healthy subjects, 34 patients with periodontitis, 33 patients with CHD, and 34 patients with periodontitis and CHD. Lipid profile and levels of MDA and C-reactive protein (CRP) were assessed. Patients in the periodontitis group (serum: 3.92 (3.7–4.4) µmol/L; salivary 1.81 (1–2.1) µmol/g protein, *p* < 0.01) and in the periodontitis + CHD (serum: 4.34 (3.7–4.8) µmol/L; salivary 1.96 (1.7–2.3) µmol/g protein, *p* < 0.001) group presented higher median concentrations of salivary and serum MAA compared to patients in the CHD (serum: 3.72 (3.5–4.1) µmol/L; salivary 1.59 (0.9–1.8) µmol/g protein, *p* < 0.01) and control group (serum: 3.14 (2.8–3.7) µmol/L; salivary 1.41 (0.8–1.6) µmol/g protein, *p* < 0.01). In univariate models, periodontitis (*p* = 0.034), CHD (*p* < 0.001), and CRP (*p* < 0.001) were significantly associated with MAA. In the multivariate model, only CRP remained a significant predictor of serum and salivary MAA (*p* < 0.001) MAA levels. Patients with periodontitis and with periodontitis + CHD presented higher levels of salivary and serum MAA compared to healthy subjects and CHD patients. CRP has been found to be a significant predictor of increased salivary and serum MAA levels.

## 1. Introduction

Periodontitis is an inflammatory and chronic multifactorial disease caused by a dysbiotic microflora of periodontal bacteria, which causes the destruction of the tooth-supporting tissues and which can finally determine tooth loss in the long-term [1]. In the USA, recent reports have been shown in which about 50% of adults aged >30 years have periodontitis and almost 10% of the world population has a severe form of periodontitis [2,3]. During the last few decades, several studies have demonstrated a strong association between periodontitis and coronary heart disease (CHD), including several conditions such as stroke, myocardial infarction, and peripheral vascular disease [4,5]. More specifically, recent systematic reviews and large cohort studies have highlighted a graded association between periodontitis, tooth loss, and increased risk of CHD [6,7].

The etiology of periodontitis comprises immunological and inflammatory processes that cause dysregulation in the host response due to the superinfection of periodontal bacteria [8]. Furthermore, periodontitis has been associated with higher serum levels of different inflammatory biomarkers, such as interleukin 6, prostaglandin, and C-reactive protein (CRP) [9].

Oxidative stress, through nitric oxide (NO), has been determined to be one of the main features in the pathogenesis of both periodontitis and CHD. Previous studies have evidenced that during active phases of periodontitis and CHD, polymorphonuclear neutrophils produce several reactive oxygen species, which can lead to further damage to periodontal and endothelial tissues [10]. NO is one of the important mediators that regulates function and vasodilatation of the endothelium because it regulates the level of inflammation in the vessels, the vascular tone, the cell proliferation, and it modulates the release of different growth factors [11]. Moreover, the augmented oxidation during periodontitis and CHD alternates some low-density lipoprotein (LDL), which can lead to the formation of lipid-laden foam cells and cholesterol in the endothelial lumen which, finally, establishes the endothelial dysfunction and atherogenesis [12].

Augmented oxidations of LDLs are also present in oral tissues [13]; more recently, several authors described increased levels of malondialdehyde acetaldehyde (MAA) adducts during periodontitis [14] and CHD [15]. MAA is a natural molecule that occurring as a lipid peroxidation product and has been shown to be strongly reactive with several lipids and proteins to form a wide range of adducts [16].

Several authors have proposed that MAA adducts contribute to aetiological mechanisms of several diseases such as CHD, hyperlipidemia, and diabetes through endothelial damage. During CHD, Anderson et al. [17] have shown higher levels of IgA and IgG antibodies to MAA-LDL, while Karvonen et al. [18] have shown that IgM antibodies to MAA-LDL present an inverse association with endothelial atherosclerosis.

During the last few decades, some authors have shown high levels of serum IgG antibodies to connect to *P. gingivalis* in atherosclerotic vascular damage [19] and some animals infected by *P. gingivalis* in a periodontitis model [20]. It has also been demonstrated that mice heat-killed with *P. gingivalis* presented increased plasma IgM to MAA-LDL and IgM antibodies to MAA-LDL virulence factor gingipain (Rgp44) [21]. Moreover, some antibodies to a virulence factor of *A. actinomycetemcomitans* heat shock protein 60 (Aa-HSP60) have been shown to react with MAA-LDL, a well-known heat shock protein molecule demonstrated as a key factor in the development of atherosclerosis [16].

Few reports have associated periodontitis and CHD with endothelial dysfunction [22,23,24]. The reduced production of NO negatively impacts the vascular endothelial cells whose impairment determines, finally, endothelial dysfunction and vasodilatation [25,26]. Hence, this has aroused interest in assessing possible oral factors that influence and regulate endothelial changes, such as subclinical signs of CHD.

In this regard, previous studies have demonstrated a strict association between NO, high serum MAA and CRP levels, and endothelial damage [27]. The local production of NO has an important role in the development and progression of periodontitis and CHD. Both increment and decrement in the production of salivary NO metabolites in gingival tissue of periodontitis patients against periodontal bacteria and periodontal tissues have been reported to be associated with impaired endothelium-dependent vasodilatation [28,29].

However, to date, the role of MAA is not well understood, as subclinical stimulus of endothelial dysfunctions in patients with periodontitis and CHD. In light of these findings, the aims of this study were to further evaluate a possible impact of periodontitis, CHD, or a combination of both periodontitis and CHD on saliva and serum MAA levels. Moreover, the association between both saliva and serum MAA levels in patients with periodontitis and with CHD was assessed, and if the salivary or serum MAA levels are mediated by serum CRP.

## 2. Results

### Study Participant

The demographic and biochemical characteristics of the recruited subjects are represented in Table 1. Patients and controls were matched for age and gender, and there were no significant differences between the distribution of education levels or median values (25%; 75% percentiles) of BMI between the groups (Table 1). Patients with CHD and periodontitis + CHD had a higher proportion of previous CVD events (stroke, atrial fibrillation, angina pectoris, and heart failure) and took more CVD drugs (statins, low-dose aspirin, antihypertensive, and beta-blockers) compared to periodontitis and healthy subjects. Increased values of hs-CRP were observed among patients with periodontitis, CHD, and periodontitis + CHD in comparison with healthy subjects (*p* < 0.001).

Table 2 shows dental and periodontal variables in patients with periodontitis, CHD, periodontitis + CHD, and controls. The median values of periodontal parameters (CAL, PD, BOP, and PI) were significantly higher, and the number of present teeth was significantly lower in patients with periodontitis and periodontitis + CHD compared with CHD and with healthy subjects (*p* < 0.001) (Table 2).

**Figure 1:**
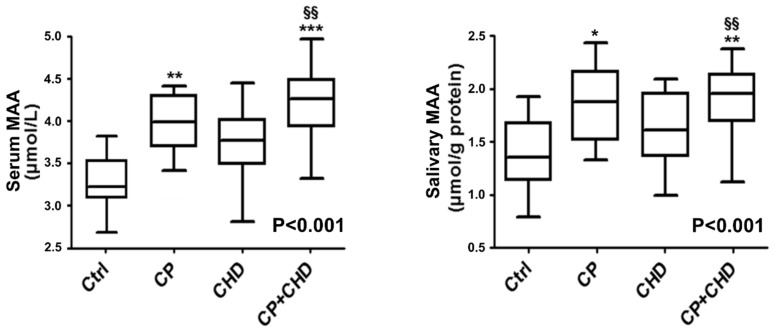
Median values (25%; 75% percentiles) of serum and salivary MAA levels in each group of subjects. * *p* < 0.05, ** *p* < 0.01, and *** *p* < 0.001 significant differences vs control subjects. ^§§^ *p* < 0.01 significant differences vs periodontitis patients. *p* < 0.001 (obtained by Jonckheere-Terpstra test). Ctrl, Healthy Controls; CP, periodontitis; CHD, Coronary Heart Disease.

Median (25th; 75th percentile) serum and salivary MAA levels are presented in Figure 1. Patients in the periodontitis group (serum: 3.92 (3.7–4.4) µmol/L; salivary 1.81 (1–2.1) µmol/g protein, *p* < 0.01) and in the periodontitis + CHD group (serum: 4.34 (3.7–4.8) µmol/L; salivary 1.96 (1.7–2.3) µmol/g protein, *p* < 0.001) presented higher median concentrations of salivary and serum MAA compared to patients in the CHD (serum: 3.72 (3.5–4.1) µmol/L; salivary 1.59 (0.9–1.8) µmol/g protein, *p* < 0.01) and control group (serum: 3.14 (2.8–3.7) µmol/L; salivary 1.41 (0.8–1.6) µmol/g protein, *p* < 0.01). Overall, a *p*-for trend was statistically significant (*p* < 0.001), denoting that serum MAA increased in healthy subjects and patients with periodontitis, CHD, and with periodontitis + CHD (Figure 1).

**Figure 2:**
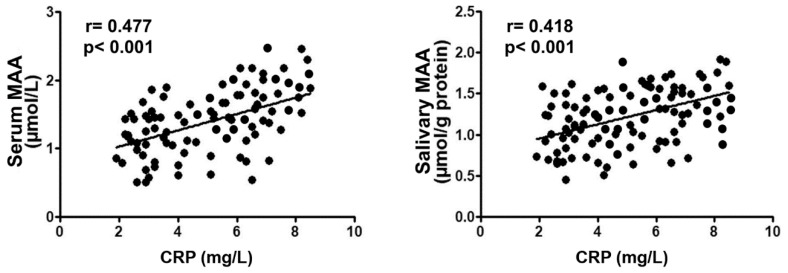
Spearman correlation between serum, salivary MAA and CRP.

There was no significant correlation between salivary and serum MAA levels (rs = 0.178, *p* = 0.095). Moreover, in all enrolled subjects, serum/salivary MAA concentrations were positively correlated (rs = 0.477, *p* < 0.001)/(rs = 0.418, *p* < 0.001) with hs-CRP levels (Figure 2).

The multivariate linear regression analysis, adjusted for several confounders and aimed at assessing the possible influence of periodontitis and CHD on serum and salivary MAA levels, showed that hs-CRP was the statistically significant predictor variable for both serum and salivary MAA levels (*p* < 0.001) (Table 3).

## 3. Discussion

This study analyzed the impact of gingival health, periodontitis, CHD, or a combination of both diseases on saliva and serum MAA levels. This study found that the presence of periodontitis in CHD patients contributed to increased levels of serum and salivary MAA and CRP levels. However, compared with periodontitis and healthy subjects, only patients with CHD and periodontitis + CHD presented significantly higher salivary and serum MAA levels, supporting the notion that periodontitis independently contributed to increased serum MAA levels in patients with periodontitis + CHD.

Our results showed that the presence of periodontal disease in patients with CHD might serve as an accelerator of activating MAA and thus act as a sub-clinical stimulus for an increased risk of endothelial damage. Recent investigations suggested that high serum MAA levels, through inactivation a specific pathway, are independent risk factors of CVD and are related to increased mortality [30]. More specifically, it has been also demonstrated that an increase in MAA levels was associated with endothelial carotid damage in patients with atherosclerosis, highlighting the inhibitory role of MAA on NO levels [31]. This inhibitory mechanism could result in an increase in collagen cross-linking and alteration in microcirculation, leading to pronounced albuminuria and the consecutively increased risk of endothelial injury [32]. The co-occurrence of periodontitis in CHD patients may be a possible pathway for the observed deterioration of endothelial function via increased MAA levels. In fact, periodontal treatment significantly decreased serum MAA levels in patients with periodontitis [33].

Moreover, periodontitis increases serum hs-CRP levels, which may mediate the increase in serum MAA levels, as shown by many groups [33,34]. Stimulating oxidative stress conditions, such as periodontitis and CVD, may have led to the high production of CRP levels, which in turn could stimulate serum and salivary MAA to protect cells from tissue damage due to oxidative stress. In accordance with our results, Reddy et al. [35], in a sample of 40 diabetic subjects with periodontitis, found that serum MAA and CRP levels were associated in a dose-dependent manner in patients with periodontitis. In the study by Reddy et al. [35], high-MAA and CRP levels were significantly associated with mean clinical attachment level (CAL), probing depth (PD), and bleeding on probing (BOP).

Furthermore, while there are some observations on the serum MAA levels as a marker for endothelial dysfunction or CVD risk, there are no reports that analyze salivary MAA levels to find out if an increase in salivary MAA levels also reflects increased serum MAA levels as a marker of endothelial damage. However, the present study did not find a statistically significant correlation between serum and salivary MAA levels; salivary MAA levels were independently influenced only by hs-CRP levels. This could be explained by the fact that the saliva levels of MAA could mainly reflect the oral production of MAA in the gingival tissue or that the salivary MAA levels may have been influenced by the saliva collection method used in the present study.

While the systemic impact of increased levels of MAA on endothelial dysfunction via decreased NO is undoubtedly proven, the effect of oral MAA activation is less clear. As a matter of fact, there are reports which show that periodontitis is positively associated with impaired salivary NO levels [28,36]. NO can be produced in the gingival tissues as part of the oral unspecific salivary antibacterial defense against anaerobic periodontopathogens bacteria [11,28,37]. However, there is no consensus about the effects of NO levels during periodontitis. Some reports showed high levels of NO synthesis and activity in the inflamed periodontal tissue [38,39], while, on the other hand, some authors reported lower salivary levels of NO in periodontitis patients [29,40]. This discrepancy may be due to the fact that some of the patients enrolled in these studies were smokers (unlike the present study all patients were not smokers), and smoking increases salivary NO levels [41]. Another explanation for the contradictory results may be due to the method of saliva collection. Moreover, we can also argue that the difference in the NO production at the periodontal level is probably different from NO in the serum: in the mouth it is antibacterial defense, whereas systemically it impacts endothelial function.

Moreover, endothelial dysfunctions in periodontitis patients with CVD could be due to a specific immunoreactive pathway in which MAA modulates a response against periodontopathic bacteria during periodontitis. It has been shown that MAA, during periodontitis, is involved in the immune response through the activated endothelium and its heat shock proteins that are present in the endothelium surface finally stimulate some IgG with particularity for host-activated antibodies [30,42,43,44]. This process modulated by MAA and other lipid biomarkers also affects the inducted defense mechanism mediated by NO, which promotes the hyperactivation of the endothelial cells that increases the risk of further infection or systemic inflammation due by periodontitis [24,45,46,47].

However, the present study presents some limitations. One of the main limitations is the cross-sectional nature of the study, which makes it difficult to separate cause and effect and the small sample size, which was due to a matching of age, gender, and education. An advantage of matching is the elimination of the effect of these confounding variables.

During the last few decades, new approaches through salivary diagnostics have been developed to evaluate the possible useful biomarkers for predicting the disease. This study indicated that patients who have periodontitis and CHD presented higher levels of serum and salivary MAA levels compared to periodontitis patients and healthy subjects, and that periodontitis and CHD act as a stimulus to increase the serum values of MAA through a pathway mediated by the hs-CRP, which also influenced salivary MAA levels. This pilot study is promising and demands further studies with a larger sample and different designs to understand better the role of MAA beyond the endothelial dysfunction in patients affected by periodontitis.

## 4. Materials and Methods

### 4.1. Study Design

Three hundred and two patients with periodontitis, CHD, and healthy controls, matched for age and gender, were selected among those who attended the school of dentistry of the University of Messina, Messina, Italy, from June 2016 to September 2018. The study was performed in accordance with the Declaration of Helsinki revised in 2016 on medical research. Ethical approval was obtained by the IRB of the University of Messina (#2016-12, 22/03/16). The study was registered at clinicaltrials.gov (NCT04030286). Written informed consent was obtained from each patient about the study characteristics and possible risks of the study. This trial followed the STROBE guidelines for the strengthening of reporting of observational studies (Table S1) [48].

Inclusion criteria for the periodontitis group were: (1) presence of at least 16 teeth, (2) a minimum of 40% of sites with clinical attachment level (CAL) ≥ 2mm and probing depth (PD) ≥ 4 mm [49]; (3) presence of at least ≥ 3 mm of crestal alveolar bone loss verified on digital periapical radiographs; and (4) presence of ≥ 40% sites with bleeding on probing (BOP) [50]. Healthy individuals presented no systemic disease, no sites with PD ≥ 4 mm or CAL ≥ 4 mm, or radiographic signs of bone loss.

Inclusion criteria for the CHD group were: at least ≥18 years old with a diagnosis of CVD, ≥50% of stenosis of at least one coronary artery by coronary angiography or coronary artery bypass surgery, or percutaneous coronary intervention [51].

The exclusion criteria for all patients were: (1) intake of contraceptives; (2) intake of immunosuppressive or anti-inflammatory drugs throughout the last three months prior to the study; (3) status of pregnancy or lactation; (4) previous history of excessive drinking; (5) allergy to local anesthetic; (6) intake of drugs that may potentially determine gingival hyperplasia such as Hydantoin, Nifedipine, Cyclosporin A, or similar drugs; (8) presence of any oral conditions (e.g., apical abscesses) that could have affected the experimental results.

After a first screening, 169 patients were excluded from the final sample because they did not meet the inclusion criteria (*n* = 131), declined to participate (*n* = 27), or did not attend the first appointment (*n* = 11). Thus, for this study, 34 patients with periodontitis, 33 patients with CHD, 34 patients with periodontitis + CHD, and 32 healthy subjects were finally enrolled.

The demographic (level of education), clinical, and medical characteristics (sex, age, body mass index, hypertension, diabetes, dyslipidemia, previous CVD events), and medications were assessed in all enrolled subjects. The presence of diabetes mellitus was based on the history of the patient or a fasting blood glucose ≥ 126 mg/dL. Body Mass Index (BMI) was estimated on the weight of the patient divided by the square of the patient’s height, i.e., kg/m^2^.

The periodontal evaluation comprised PD, CAL, BOP, and plaque score (PI) [52,53]; the presence of bleeding was recorded up to 30 s after probing. CAL was recorded as PD plus recession with the cementoenamel junction as a reference for CAL measurements. All clinical periodontal parameters were recorded at six sites per tooth on all teeth present, excluding third molars.

Duplicate full-mouth periodontal evaluations were performed by two calibrated examiners not involved in the subsequent data analysis with a manual periodontal probe (UNC-15, Hu-Friedy, Chicago, IL, USA). In the case of discordant measurements ≥2 mm PD, a new clinical assessment was performed. Intra- and inter-examiner reproducibility of CAL was assessed from randomly selected patients. The inter-examiner reliability test resulted in an agreement of 86.1% (*k* = 0.58) for the outcome CAL. The intra-examiner agreement was evaluated by the measurement of Cohen’s k coefficient, which was 0.861, and which equaled a high degree of reliability. The kappa coefficient was also calculated for the measurements taken at each follow-up session, and an acceptable degree of reliability (ICC = 0.812) was established for every examination.

A power analysis was performed to calculate the minimum sample size required. The sample size was established considering a number of groups equal to 4, an effect size of 0.35 for MAA (that represented the primary outcome variable), an expected standard deviation of 1.5 [54], a two-sided significance level of 0.05 and a power of 80%. It was determined that approximately 27 patients per group would be needed. Thus, we estimated that overall the 130 subjects were needed to ensure a power level of 77%.

### 4.2. Serum and Salivary MAA Measures

Fasting samples were collected in all subjects between 8:00 am and 10:00 am. Participants were asked to refrain from eating, drinking, chewing gum, tooth brushing, as well from using any mouthwashes in the last 12 h before the sampling.

The venous puncture was performed, and blood samples were collected, cooled on ice immediately, and centrifuged at 4 °C (800× *g* per 10 min). To collect saliva, subjects were asked to chew on a cotton roll for 2 min, and saliva samples were collected using Salivette collection devices (Sarsted, Verona, Italy) and immediately centrifuged at 4 °C (1000× *g* per 2 min). Serum and saliva samples were stored at −20 °C until analysis.

Levels of MAA were assessed by double-antibody sandwich enzyme-linked immuno-sorbent one-step process assay (ELISA), using ready to use in vitro kits as per manufacturer’s protocol (Shanghai Qayee Biotechnology Co., Ltd., Shanghai, China). In fasting conditions, reference ranges are 0.3–1.3 µM for MAA, and hs-CRP were assessed by a commercially available nephelometric assay. An hs-CRP level higher than 3 mg/L was associated with increased risk of CVD. Plasma lipids and glucose were determined by routine methods.

### 4.3. Statistical Analysis

The numerical data were expressed as median, 25%, and 75% percentile, and categorical variables as number and percentage. Since examined variables did not present normal distribution as verified by a Kolmogorov–Smirnov test, the analysis was performed by nonparametric tests. In particular, the Kruskal–Wallis test was applied in order to compare the four groups with regards to all numerical variables and the Mann–Whitney test was applied in order to perform two-by-two comparisons between groups. For these multiple comparisons, Bonferroni’s correction was applied, for which the significant alpha level 0.050 was divided by the number of possible comparisons (*n* = 6), so the “adjusted” significance level for this analysis was equal to 0.050/6 = 0.008. A *p*-trend was performed with the Jonckheere-Terpstra Test for serum and salivary MAA levels to assess if the MAA levels significantly increase with healthy, periodontitis, CHD, and with periodontitis and CHD. The Spearman correlation test was applied to assess the existence of any significant interdependence between hsCRP and serum, respective saliva MAA.

In all enrolled subjects, univariate and multivariate linear regression models were performed in order to assess the dependence of salivary and serum MAA levels on potentially explicative variables such as age, gender, education, socioeconomic status (SES), BMI, CRP, triglycerides, and total cholesterol. In the final multivariate model, only age, gender, and education SES were included as confounders and tests were carried out to analyze if periodontitis, CHD, and hs-CRP influenced serum MAA. The same analysis was performed for salivary MAA as an outcome.

Statistical analyses were performed using SPSS 22.0 for Windows package. A *p*-value < 0.05 was considered statistically significant.

## Figures and Tables

**Table 1:** Individual characteristics and biochemical parameters of recruited subjects. Data are expressed as median (25th; 75th percentiles) or number with percentage. * *p* < 0.001 and ** *p* < 0.001 significant differences vs healthy subjects calculated by the Mann–Whitney test. ^§§^ *p* < 0.001 significant differences vs periodontitis patients calculated by the Mann–Whitney test. *p* < 0.007 significant differences vs CHD patients calculated by the Mann–Whitney test. CHD, coronary heart disease; CVD, cardiovascular disease.

Clinical Features	Controls (*n* = 32)	Periodontitis (*n* = 34)	CHD (*n* = 33)	Periodontitis + CHD (*n* = 34)
Age (years)	58 (54; 60)	57 (52; 59)	55 (49; 58)	56 (50; 59)
Gender (male/female)	16/16	16/18	17/15	17/17
Education level				
Primary school, *n* (%)	11 (34.3)	12 (35.3)	11 (33.3)	13 (38.3)
High school, *n* (%)	13 (40.6)	14 (41.2)	15 (45.5)	13 (38.3)
College/university, *n* (%)	8 (25)	8 (23.5)	7 (21.2)	8 (23.5)
Body mass index (kg/m^2^)	27.2 (25.6; 28.7)	24.5 (23.4; 28.4)	26.4 (23.2; 27.8)	22.8 (20.6; 26.3)
Fasting glucose (mg/dL)	89.1 (86.9; 91.8)	89.6 (81.8; 129.3)	89.1 (86.9; 132.4)	91.3 (87.8; 128.6)
Current smokers, *n* (%)	2 (6.2)	3 (8.8)	3 (9)	7 (8.8)
Comorbidities				
Diabetes, *n* (%)	-	4 (11.8) **	5 (15.1) **	4 (11.8) **
Previous CVD				
Atrial fibrillation, *n* (%)	-	-	6 (18.1)**^,§§^	10 (29.4) **^,§§^
Angina pectoris, *n* (%)	-	-	17 (51.5)**^,§§^	18 (53) **^,§§^
Stroke, *n* (%)	-	-	9 (27.3) **^,§§^	11 (32.3) **^,§§^
Heart failure, *n* (%)	-	-	10 (30.3) **^,§§^	11 (32.3) **^,§§^
Drug treatment of CVD				
Antihypertensive, *n* (%)	-	-	14 (42.4) **^,§§^	15 (44.1) **^,§§^
Statins, *n* (%)	-	-	13 (39.4) **^,§§^	13 (38.2) **^,§§^
Low-dose aspirin, *n* (%)	-	-	12 (36.4) **^,§§^	12 (35.3) **^,§§^
Beta blockers, *n* (%)	-	-	13 (39.4) **^,§§^	14 (41.2) **^,§§^
hs-CRP (mg/L)	2.7 (2.3; 3.0)	3.5 (3.1; 4.1) *	6.1 (5.4; 6.3) **	6.8 (6.1; 8) **^,§§,#^
Total cholesterol (mg/dL)	161 (125; 186)	171 (144; 197)	177 (153; 198)	174 (155; 201)
Triglycerids (mg/dL)	137 (107; 145)	114 (59; 122)	141 (112; 168)	143 (111; 159)

**Table 2:** Clinical dental variables of recruited subjects. Data are expressed as median (25th; 75th percentile). CAL, clinical attachment level; PD, probing pocket depth; BOP, bleeding on probing; PI, Plaque index. ** *p* < 0.001 significant differences vs control subjects calculated by the Mann–Whitney test. ^§§^ *p* < 0.001 significant differences vs periodontitis patients calculated by the Mann–Whitney test. ^##^ *p* < 0.001 significant differences vs. CHD patients calculated by the Mann–Whitney test.

Periodontal Parameters	Controls (*n* = 32)	Periodontitis (*n* = 34)	CHD (*n* = 33)	Periodontitis + CHD (*n* = 34)
N of teeth	25 (24; 27)	17 (15; 19) **	22 (21; 24) **^,§§^	18 (15; 19) **^,##^
CAL (mm)	1.2 (1; 1.3)	3.8 (3.2; 4.2) **	2.3 (2.2; 2.6) **^,§§^	3.8 (3.7; 4.6) **^,##^
CAL 4–5 mm (% sites)	-	39.1 (37.3; 41.8) **	-	42.2 (37.4; 49.1) **^,##^
CAL ≥6 mm (% sites)	-	18.2 (18.7; 21.4) **	-	18.8 (16.4; 24.5) **^,##^
PD (mm)	1.6 (1.2; 2.1)	4.3 (4.1; 4.8) **	2.1 (1.7; 2.3) **^,§§^	4.1 (4.0; 4.8) **^,##^
PD 4–5 mm (% sites)	-	41.8 (40.7; 44.6) **	-	43.8 (40.4; 55.7) **^,##^
PD ≥6 mm (% sites)	-	21.5 (18.9; 23.6) **	-	25.3 (21.4; 27.5) **^,§§,##^
BOP (%)	9.9 (8.8; 11.4)	45.9 (45.7; 51.8) **	11.5 (14.5; 18.6) **^,§§^	47.5 (45.6; 55.2) **^,§§,##^
PI (%)	10.1 (9.6; 11.2)	35.6 (33.5; 36.2) **	15.2 (14.5; 16.7) **^,§§^	33.5 (32.5; 32.9) **^,##^

**Table 3:** Uni and multivariate linear regression model for serum and salivary MAA levels in all enrolled patients. Age was included as a continuous variable. For periodontitis and CHD, controls served as reference, and, for gender, males served as reference. For education, primary school served as a reference.

		Univariate			Multivariate		
	Variable	B	95% CI	*p*	B	95% CI	*p*
**Serum MAA Levels**	CHD	0.384	0.221; 0.569	**<0.001**	0.075	−0.356; 0.531	0.665
	Periodontitis	0.221	0.015; 0.412	**0.041**	0.161	−0.039; 0.355	0.133
	Hs-CRP	0.115	0.071; 0.156	**<0.001**	0.133	0.056; 0.178	**<0.001**
	Age (years)	−0.022	−0.35; 0.004	0.088	−0.031	−0.208; 0.204	0.416
	Female gender	0.156	−0.035; 0.358	0.156	0.145	−0.121; 0.356	0.065
	Education SES	−0.071	−0.202; 0.054	0.289	−0.075	−0.156; 0.31	0.132
**Salivary MAA Levels**	CHD	0.281	0.134; 0.431	**<0.001**	−0.051	−0.431; 0.356	0.769
	Periodontitis	0.073	−0.089; 0.232	0.456	0.004	−0.145; 0.181	0.782
	Hs-CRP	0.089	0.043; 0.131	**<0.001**	0.096	0.041; 0.156	**<0.001**
	Age (years)	−0.012	−0.021; 0.007	0.346	0.004	−0.012; 0.023	0.754
	Female gender	0.047	−0.131; 0.223	0.462	0.089	−0.089; 0.312	0.278
	Education SES	−0.095	−0.183; −0.004	**0.048**	−0.89	−0.056; 0.332	**0.039**
	Serum MAA	0.166	−0.031; 0.338	0.079	−0.045	−0.234; 0.136	0.599

## Supplementary Materials

Supplementary materials can be found at https://www.mdpi.com/1422-0067/20/23/6061/s1.
